# Brown Syndrome: Features and Long-term Results of Management

**DOI:** 10.14744/bej.2021.35693

**Published:** 2021-09-27

**Authors:** Ahmet Alperen Koc, Ebru Demet Aygit, Asli Inal, Bulut Ocak, Ceren Gurez, Sibel Ahmet, Bugra Duman, Birsen Gokyigit

**Affiliations:** 1. Department of Ophthalmology, Istanbul Aydın University Medical Park Florya Hospital, Istanbul, Turkey; 2. Department of Ophthalmology, Istanbul Beyoglu Goz Training and Research Hospital, Istanbul, Turkey; 3. Department of Ophthalmology, Istanbul Catalca State Hospital, Istanbul, Turkey; 4. Department of Ophthalmology, Istanbul Basaksehir Cam ve Sakura City Hospital, Istanbul, Turkey

**Keywords:** Abnormal head posture, Brown syndrome, limitation of elevation in adduction, ocular movements

## Abstract

**Objectives:**

The goal of this study was to evaluate surgical techniques and outcomes in patients with Brown’s syndrome.

**Methods:**

A retrospective review was conducted of patients who underwent surgery of the superior oblique (SO) muscle between 2003 and 2011 at a referral center.

**Results:**

In all, 190 patients (111 female and 79 male) with an age range of 4-50 years were included in the study. The right eye was affected in 98 patients, and the left eye in 92 patients. Abnormal head posture (AHP), ocular movement (OM), and hypotropia were assessed. The greatest improvement of AHP was seen following an SO temporal tenotomy (91%). Patients with a -4 limitation achieved full OM after a SO temporal tenotomy.

**Conclusion:**

Temporal tenotomy provided the best improvement in limitation of elevation in adduction.

## Introduction

Brown’s syndrome is a form of restrictive strabismus first described by Brown in 1905. It is characterized by a restriction of the superior oblique trochlea-tendon complex and patients have limited elevation in adduction that can be detected clinically as compensatory head posture. This syndrome may present in congenital, acquired, constant, or intermittent forms (1). Surgery is frequently indicated in the presence of strabismus or abnormal head position with Brown’s syndrome (2). The surgical procedure used to address Brown’s syndrome is based on the tight or stiff superior oblique muscle (SOM)-tendon complex (3). Tenotomy and tenectomy are the most popular surgical treatments for Brown’s syndrome.

The aim of this study is to determine the demographic characteristics of 190 patients who were diagnosed with Brown syndrome, also describe surgical preferences and the long-term outcomes of surgery in Brown’s syndrome.

## Methods

This study retrospectively analyzed the medical charts of 190 patients that had been followed for Brown’s syndrome and subsequently performed surgery of the SO muscle, as indicated by strabismus or abnormal head posture between 2003 and 2011 in the Beyoglu Goz Training and Research Hospital, Strabismus Department, Istanbul, Turkey. The study was conducted in accordance with the ethical standards of the Declaration of Helsinki.

In this study, patients with previous eye muscle surgery, any ocular or systemic disease, or lack of cooperation during ophthalmologic examination (e.g., alternate prism cover testing and ocular movement) were excluded from the study.

All patients had a routine ophthalmologic and orthoptic evaluation, including best-corrected visual acuity (BCVA), the angle of deviation, ocular motility testing at nine diagnostic gaze positions, abnormal head position, and stereoacuity before and after surgery, and during followed period. Demographic features, such as onset age, gender, refractive situation, and ocular case history, were recorded from medical charts.

The onset age of apparent head position was defined as the age at which the parents/caregiver or a physician first recognized ocular misalignment. The values recorded by the parents/caregiver were later checked using old photographs of the patients. The BCVA was checked with Snellen charts. Cover/uncover test and alternate prism cover test at 1/3 and 6 m (Krimsky and Hirschberg test in patients without cooperation) were conducted on all patients, who wore their glasses when necessary in nine different gaze positions. Sensory status was also determined with a Titmus stereo test (Stereo Optical Co., Inc., Chicago, IL) at 1/3 m. The restriction of the SOM was estimated from −1 to −4. Stability of the head position of the patients in primary gaze was obtained and examination ensured. We defined Brown syndrome as a −1–−4 limitation of elevation in adduction with or without hypotropia in primary position and a downward displacement of the affected eye on adduction. Surgery was recommended when the abnormal head position and the vertical deviation were stable, over 2–3 visits. Surgeries were performed by one surgeon (BG).

All testing were performed in the post-operative period and were followed up every 2 weeks for 2 months, monthly for a minimum of 6 months, and at differing intervals after that.

Surgeries were done if there was a prominent abnormal head position, and hypotropia increased to 10 prism diopter (PD) in the primary position, these signs had been found for 4 years.

After the post-operative evaluation, we defined successive surgical treatment to achieve a hypotropia of 10 PD or less in the primary position and no (or less 10°) abnormal head position.

For statistical analysis, p<0.05 was considered as statistically significant.

Data were analyzed using the Wilcoxon and McNemar tests.

## Results

In this study, there were 190 patients, 111 (59%) of them were female. The mean age was 9 years (range: 4–50 years), and 98 (51%) right eyes and 92 (49%) left eyes were involved. The follow-up period was 21 months (from 6 months to 19 years). Demographic futures are summarized in Table 1.

**Table 1.** Demographic features of patients

**Characteristics**	**Patients (n=190)**
Age	9 (4-50 years)
Gender	
Female	111 (59%)
Male	79 (41%)
Laterality	
Right	98 (47%)
Left	92 (43%)
Follow-up	21 months (from 6 months to 19 years)
Ambylopia	43 (22.6%)
AHP	94 (49%)
Hypotropia	50 (25%)

AHP: Abnormal Head Position.

Historical features included consanguineous marriage (32%), positive familial history (8%), febrile convulsion (3.7%), trauma (1.5%), and prematurity (0.5%) (Table 2). Orthotropia was present in 98 (51%) patients, esotropia in 36 (19%) patients, right hypotropia in 30 (15%) patients, left hypotropia in 20 (10%) patients, and exotropia in 6 (3%) patients.

**Table 2.** History of patients

	**n (%)**
Consanguineous marriage	62 (32)
Positive familial history	17 (8)
Febrile convulsion	7 (3.7)
Trauma	3 (1.5)
Prematurity	1 (0.5)

Cycloplegic refraction of patients, mean spherical equivalent was determined +1.25±1.8 in the right eye and +1.6±1.8 in the left eye. Amblyopia was found in 43 (22.6%) patients.

Head adaptation was consistent with Brown’s syndrome; head tilt to the affected side, a face turn to the normal side, and chin elevation was seen in 94 (49%) patients.

The clinical findings of Brown’s syndrome patients are summarized in Table 3. Limited elevation in adduction was demonstrated in all patients (100%). The head posture was presented in 94 (49%), V-pattern in 10 (0.5%), and down-shoot in adduction in 6 (0.3%) patients.

**Table 3.** Clinical features of Browne Syndrome

	n (%)
Restriction of elevation in adduction	190 (100)
Abnormal head position	94 (49)
Orthotropia	98 (51)
Hypotropia	50 (25)
V-patern	10 (0.5)
Downshoot	6 (0.3)

Patients were scored from the severity of their restriction of elevation in adduction. Eighteen patients scored −5, 88 patients scored −4, 71 patients scored −3, and 13 patients scored −2 restriction of elevation in adduction (Table 4).

**Table 4.** Distribution of restriction of elevation in adduction

-5	-4	-3	-2
	9.5% (18)	46.3% (88)	37.4% (71)	6.8% (13)

**Figure 1.**
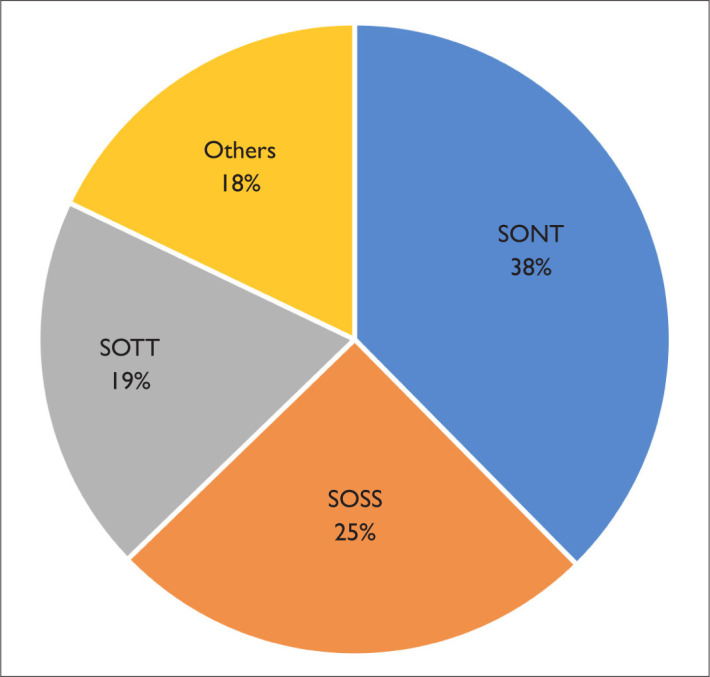
Distribution of performed Surgery. SONT: Superior oblique nasal tenetomy. SOSS: Superior oblique suture spacer. SOTT: Superior oblique tenectomy. OTHERS: Superior oblique silicone spacer (4.4%).Superior oblique intracapsuler tenetomy (3.7%). Superior oblique Z spacer (2.8%). Inferior oblique plication (2.4%). Thinning of the superior oblique tendon (1%). Superior rectus FADEN (0.2%).

First choice of surgical technic in patients who underwent surgery was superior oblique tenotomy (total 56.7%, 62 patients [48.1%] nasal tenotomy and 11 patients [8.6%] temporal tenotomy). Second, 33 (25.6%) of 129 patients were performed superior oblique tendon suture spacer surgery. Posterior tenectomy procedure was used 10 (5.3%) patients in our study. A silicone expander into the SO tendon technic was performed in 8 (4.21%) patients. Details of performed surgery are suggested in Figure 1.

Postoperatively, AHP was detected in only 28 patients (23.3%) (p<0.05), the mean LEA improved from −3.58±0.75 to −0.90±0.96 (p<0.05), and the mean hypotropia decreased from 13.15±13.41 to 4.25±5.75 PDs (Table 5). Results were compared the frequently performed three surgical technic for the AHP, LEA, and hypotropia; statistically significance was not found among three surgeries.

**Table 5.** The effects of surgical techniques on AHP and LEA

		**Preoperative**		**Postoperative**		
		**n**	**%**	**n**	**%**	**p**
Superior oblique nasal tenetomy					
AHP	(+)	44	57.1	10	13.0	<0.05
	(-)	33	42.9	67	87.0	
Superior oblique temporal tenectomy					
AHP	(+)	7	30.4	2	8.7	<0.05
	(-)	16	69.6	21	91.3	
Superior oblique suture spacer					
AHP	(+)	30	57.7	10	19.2	<0.05
	(-)	22	42.3	42	80.8
Superior oblique tenectomy					
AHP	(+)	5	50.0	2	20.0	<0.05
	(-)	5	50.0	8	80.0	
Superior oblique silicone spacer				
AHP	(+)	2	25.0	1	12.5	>0.05
	(-)	6	75.0	7	87.5	

		**Preoperative**		**Postoperative**		
		**Mean±SD**	**Median**	**Mean±SD**	**Median**	

Superior oblique nasal tenetomy						
LEA		3.58±0.73	-4.00	0.84±0.95	-1.00	<0.05
Superior oblique temporal tenectomy						
LEA		3.61±0.99	-4.00	0.83±0.78	-1.00	<0.05
Superior oblique suture spacer						
LEA		3.48±0.67	-3.00	0.88±0.94	-1.00	<0.05
Superior oblique tenectomy						
LEA		3.60±0.84	-4.00	1.40±1.26	-1.00	<0.05
Superior oblique silicone spacer						
LEA		4.00±0.53	-4.00	1.00±0.53	-1.00	<0.05

AHP: Abnormal head position; LEA: Limitation of elevation in adduction; SD: Standard deviation.

Post-operative SO paresis that occurred in 25 patients was due to a tenotomy, suture spacer, and tenectomy in 20 (80%), 3 (12%), and 2 (8%) patients, respectively. SO paresis was significantly higher after a tenotomy (p<0.05).

## Discussion

In this study, we assessed clinical features of followed patients with Brown’s syndrome and the various surgical approaches for Brown’s syndrome and their long-term results.

The clinical characteristics of our patients were similar to those reported in the literature. In our study, there was a higher incidence of the Brown’s syndrome in females (59%) than in males (41%). In Dubinsky-Pertzov’s study, 9 patients were men (52.9%) and 8 were women (47.1%), and Sekeroglu et al. (5) reported 18 (40.9%) men and 26 (59.1%) women patients (4). Various rates have been found of the involvement of the eye; of 60% left eye (6) versus 40% right eye, 19 (43.2%) right eye versus 25 (56.8%) left eye (5), and nine left eye affected of 15 patients (7). In our study, 98 (47%) right eyes were affected versus 92 (43%) left eyes. About 10% affected bilaterally.

Our surgical indication criteria were the same as those described by Von Noorden (8). Von Noorden’s study proposed that surgery must be performed if there is a hypotropia in the primary position, a significant anomalous head posture, and when Brown’s syndrome is congenital and constant. Several procedures can be used to improve Brown’s syndrome. Successfully SOM tenotomy was proposed by Crawford in 1976 (9). Later, Von Noorden and others demonstrated that a SOM tenotomy is the most effective surgery for Brown’s syndrome (8, 10). In our study, tenotomy was the most frequently performed surgical procedure (52.6% tenotomy, 40.5% nasal tenotomy, and 12.1% temporal tenotomy). Inferior oblique muscle overaction (IOOA) frequently occurred after a tenotomy or tenectomy procedure of SO (11). Post-operative SO palsy and IOOA occurred in 20% of our study group.

Gräf et al. (12) performed SO recession surgery and found that recession is an effective and safe surgical procedure for Brown’s syndrome but that the success rate of this approach depends on the disease etiology. Astle et al. (13) described the cases of five patients with inferior oblique muscle palsy and three patients with Brown’s syndrome and proposed that recession of the superior oblique tendon (12–14 mm) is successful and reversible. In this study, the recession procedure of the superior oblique tendon was not used.

In our study, the second most performed surgical procedure was superior oblique tendon suture spacer using non-absorbable suture (27.4%). This procedure was performed according to the method devised by Suh et al. (14), in which the SO suture spacer procedure was performed on patients with moderate and severe forms of the congenital unilateral Brown’s syndrome, resulting in significant improvements in deviation and abnormal head position. In our study, we also achieved significant improvement using the SO suture spacer procedure.

In 1991, Wright designed a procedure for lengthening the short SOM tendon. This procedure retains the posterior insertion of the SO tendon, and a silicone expander is inserted into the SO tendon (15). Although this silicone expander provides a controlled weakening, the procedure is more difficult, requires more experience, and takes longer to perform than a tenotomy. Post-operative SO paresis, adhesion syndrome, granuloma, ocular inflammation, and irritation and the possibility of silicone expander extrusion were detected (16). Wright et al. (17) reported a 30% incidence of post-operative SO paresis. However, Stager et al. (18) reported less overcorrection (12.5%) and found that fewer patients require a second operation when using the silicone expander procedure. In our series, we performed silicone expander procedure in 8 (4.21%) patients, and we have not observed SO paresis or adhesion syndrome. In one eye developed band extrusion of the silicone implant postoperatively at 1-year follow-up.

Lengthening of SOM is another procedure for a weakening of SOM. Lengthening of SO tendon can make Z-tenotomy procedure. Bardorf et al. (19) study showed that Z-tenotomy procedure is highly effective for the SOM normalization and reduce of A-pattern strabismus. In Moghadam et al. (20) study, they used procedure similar to the Z-tenotomy in patients with severe type of congenital Brown syndrome. They reported that split tendon lengthening of SOM (10 mm) procedure eliminated face turn and improved limitation of elevation in most patients. Talebnejad et al. (21) used to Achilles tendon for superior oblique tendon expansion. They revealed that acceptable short-term results were obtained. This study was recommended with more cases and longer follow-up for the stability of the clinical results and the long-term effectiveness and safety of this procedure (21). Jenthani et al. (22) have worked to effect on the torsion of superior oblique split lengthening (SOSL). They were showed that the SOSL reduces intorsion post-surgery and a valuable procedure in torsion needs to be corrected (22). Achilles tendon was not used in our case series.

SO posterior tenectomy for Brown’s syndrome is advantageous because it provides a controlled weakening and low incidences of post-operative SO palsy. Kaiser et al. (23) and Velez et al. (24) found that this procedure was useful in patients who had Brown’s syndrome. Although the patients of the Velez et al. study did not develop SO palsy, these patients did have a small vertical deviation in the primary position (24). The posterior tenectomy procedure was used to treat 10 (5.3%) patients in our study. Two patients had abnormal head position in the post-operative period. Our experience with posterior tenectomy, its less effective among the other weakening procedures.

Following Brown’s syndrome surgery, patients have a high risk of developing superior oblique palsy, which requires secondary surgery (8, 25). In 1987, Parks et al. (26) reported the results of simultaneous superior oblique tenotomy and 14 mm inferior oblique recession for true Brown’s syndrome. These authors found that simultaneous surgery provided the most reliable results in the treatment of Brown’s syndrome and recommended this procedure for all patients selected to undergo surgery for true Brown’s syndrome. Sprunger et al. (27) reviewed the cases of 38 Brown’s syndrome patients treated between 1965 and 1989. These authors do not advocate simultaneous surgery for iatrogenic superior oblique palsy as an initial procedure for Brown’s syndrome because, among their patients, there were no cases of superior oblique palsy at 1-year follow-up. We do not use simultaneous surgery in our clinic and, similar to Sprunger et al., we find that patients with Brown’s syndrome require an average of two operations to achieve an acceptable result.

There are some limitations to our study. First, its retrospective design is a limitation. Second, the sensory features have not been evaluated. Third, patients in the surgical groups were not equally distributed, and some patients did not come to follow-up. Nevertheless, the larger sample size and relatively long follow-up time are strengths of our study.

## Conclusion

Various surgical techniques can be successfully applied to Brown’s syndrome. The absence of vertical tropia (squint) in the primary position or anomalous head posture was success criteria and achieved acceptable results. Patients should be followed for amblyopia and sensory features in the post-operative period.

## Disclosures

### Ethics Committee Approval:

Retrospective study.

### Peer-review:

Externally peer-reviewed.

### Conflict of Interest:

None declared.

### Authorship Contributions:

Involved in design and conduct of the study (AAK, BD); preparation and review of the study (BG, EDA); data collection (SA, OBO); and statistical analysis (AHİ, BG).
